# A Rare Case of Metaplastic Breast Carcinoma

**DOI:** 10.7759/cureus.92086

**Published:** 2025-09-11

**Authors:** Hannah C Cobb, Komalpreet Kaur, Ashlyn S Everett, Richard Virgilio

**Affiliations:** 1 Oncology, Edward Via College of Osteopathic Medicine, Auburn, USA; 2 Radiation Oncology, Edward Via College of Osteopathic Medicine, Auburn, USA; 3 Clinical Affairs, Edward Via College of Osteopathic Medicine, Auburn, USA

**Keywords:** aggressive breast cancer, breast cancer research, breast radiation, metaplastic breast cancer, radiation and medical oncology

## Abstract

Metaplastic breast cancer (MBC) is a sporadic and highly aggressive form of breast cancer characterized histologically by a group of highly heterogeneous cells. Typical presentation is marked by a large palpable breast mass without lymph node involvement. MBC is a common cause of triple-negative breast cancer. In this case, we describe a 65-year-old woman who presented with a palpable breast mass and associated inflammatory breast changes. Following a thorough work-up with a breast ultrasound, bilateral breast magnetic resonance imaging (MRI), and an image-guided needle biopsy, the patient was found to have triple-negative MBC, with squamous differentiation. This case highlights the current treatment methods employed for MBC and displays the challenging therapeutic task encountered when managing this disease. This report aims to emphasize the relevance of this cancer type and to encourage further research toward the development of disease-specific treatment guidelines.

## Introduction

Metaplastic breast cancer (MBC) is an extremely rare subtype of invasive breast cancer, with a characteristically poor response to existing treatment options and a limited survival rate. Contrary to other invasive breast cancers, MBC is marked by a unique heterogeneous cell population including spindle cells, squamous cells, and mesenchymal tissue differentiation [1]. Owing to its aggressive nature, MBC commonly presents with a large tumor and locally advanced disease at the time of diagnosis [2]. While most MBC cases are triple-negative, the treatment response varies from other triple-negative breast cancers in that it typically shows limited response to standard cytotoxic agents, resistance to chemotherapeutic efforts, and poorer prognosis when compared to triple-negative breast cancer [1]. Largely due to the rarity of this tumor type, targeted and effective treatment interventions are limited. The goal of this report is to increase relevance and promote future research efforts aimed at improving disease prognosis. There are currently six distinct subtypes of MBC as defined by the 2019 World Health Organization classification of breast tumors. These subtypes include the following: low-grade adenosquamous carcinoma (LGAC), squamous cell carcinoma (SCC), spindle cell carcinoma (SpCC), fibromatosis-like metaplastic carcinoma (FLMC), metaplastic carcinoma with mesenchymal differentiation (MCMD), including chondroid, osseous, rhabdomyoid, and neuroglial components, and mixed metaplastic carcinoma (MMC) [3]. Each of these subtypes consists of unique morphological traits that guide clinical treatment strategies. One recent study consisting of 132 patients revealed the heterologous mesenchymal subtype to be associated with the highest five-year overall and breast cancer-specific survival rates, whereas those with the squamous subtype were determined to have inferior survival [4]. While the exact etiology of MBC is largely unknown, it is understood to be multifactorial, involving genetic, environmental, and molecular alterations. Furthermore, several studies highlight a key molecular transdifferentiation process at the center of this disease [5-7]. 

Epithelial-mesenchymal transition (EMT) has been identified as a correlating factor contributing to the chemoresistance, aggressive nature, and highly metastatic potential of MBC. Throughout this process, epithelial cells lose their characteristic features and subsequently morph into mesenchymal-like cells. Consequently, cells that were previously tightly organized and attached with intercellular junctions now become motile, elongated, and invasive. The main inducers behind this process are thought to be epithelial-related transcription factors (EMT-TFs), known as ZEB1 and ZEB2, Snail1 (Snail) and Snail2 (Slug), and Twist [5]. Following EMT, cells express distinctive mesenchymal cell markers, including vimentin, fibronectin, and N-cadherin [8]. 

MBC is predominantly triple-negative, lacking the expression of receptors for estrogen, progesterone, and human epidermal growth factor receptor 2 (HER2)/neu. Without these clinical targets, current therapeutic strategies are limited to surgery, chemotherapy, and radiotherapy. Employing a combination of these therapies has been shown to provide superior results compared to using any one treatment individually [2]. When compared to non-metaplastic triple-negative breast cancer, MBC patients were observed to have a decreased five-year overall survival (OS) (65.2% vs. 60.5%; p<0.001) [9].

In this report, we describe a patient presenting with a painful, palpable breast mass and associated erythema, nipple retraction, and skin dimpling. The presence of skin involvement represents the propensity of MBC to present at advanced stages. Incorporating timely screening methods and patient education on self-examinations into clinical practice is essential, as early detection can significantly enhance disease outcomes and reduce morbidity and mortality rates. The resistant characteristics of MBC have sparked increasing interest in developing innovative targeted therapies. 

## Case presentation

**Figure 1:**
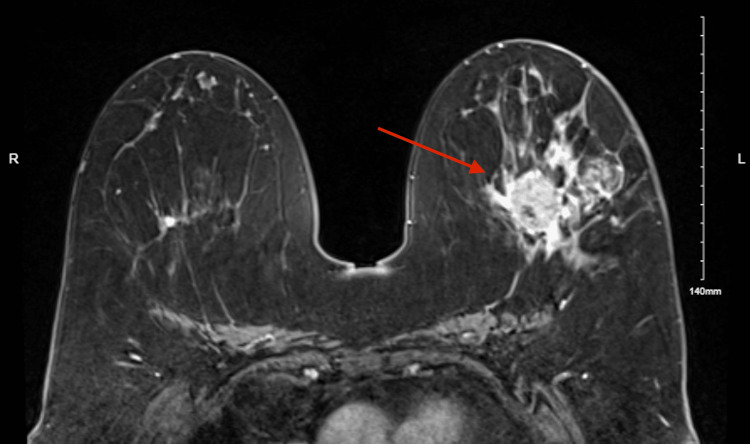
Pre-treatment contrast-enhanced breast MRI This image depicts a contrast-enhanced breast MRI that was obtained before the initiation of treatment. The red arrow indicates a large, irregular mass with spiculated margins in the left breast. MRI: magnetic resonance imaging

A 65-year-old woman with a past medical history of hypertension, hypothyroidism, and anxiety presented with a palpable left breast mass of two months' duration, associated breast pain, extensive erythema, and nipple retraction. She denied any nipple discharge or pruritus upon initial presentation. The patient had no known family history of benign breast mass or breast malignancy. An exam of the right breast demonstrated an absence of irregularity without the presence of breast pain, erythema, or nipple retraction. Subsequent diagnostic mammogram revealed a 3.8 cm mass with areas of enhancement extending a total of 7.1 cm anterior-posterior in the posterior third of her left breast. This lesion is depicted clearly in Figure 1 as a left breast mass with an irregular shape, spiculated margins, and associated heterogeneous calcifications. A magnetic resonance imaging (MRI)-guided needle biopsy was performed of this mass, followed by histological evaluation. The final pathology report revealed grade 2, triple-negative, metaplastic carcinoma with squamous differentiation. 

**Figure 2:**
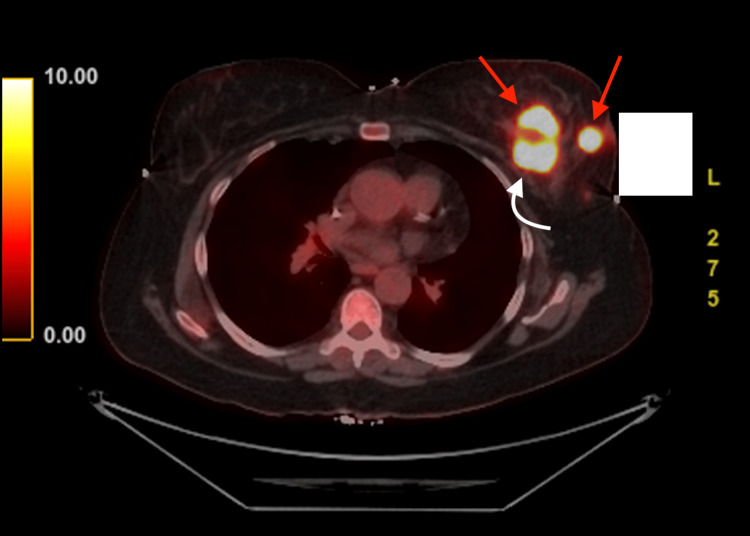
Axial fused PET-CT This image depicts an axial fused PET-CT demonstrating multiple hypermetabolic lesions (red arrows) within the left breast and extending to the chest wall (white arrow). PET-CT: positron emission tomography-computed tomography

Additionally, this patient underwent computed tomography (CT) scanning of the abdomen and pelvis. The results of this scan were negative for evidence of metastatic disease. Treatment for this patient's breast cancer was initiated with neoadjuvant therapy consisting of pembrolizumab, paclitaxel, carboplatin, doxorubicin, and cyclophosphamide. Ultimately, the decision was made to discontinue this regimen after only four months of treatment due to the radiological and clinical evidence of disease progression. A repeat physical exam revealed evidence of cutaneous metastasis involving the left chest wall and axilla. In addition to the physical findings, a positron emission tomography (PET)-CT performed at this time in the axial plane confirmed disease progression. Avid 2-deoxy-2-[18F]fluoro-D-glucose (FDG) uptake can be seen, revealing multiple hypermetabolic lesions in Figure 2, thereby affirming the formidable nature of this disease. 

With clear evidence of treatment resistance, the patient underwent a unilateral left mastectomy and sentinel lymph node biopsy according to guidelines. Pathologic staging showed ypT3(m)N0M0, indicating post-neoadjuvant therapy: T3 signifies a primary tumor larger than 50 mm with multiple foci, N0 denotes no lymph node involvement, and M0 indicates no distant metastasis. Two months after the surgical intervention, followed by appropriate wound healing, the patient began adjuvant radiation therapy to the ipsilateral chest wall and entire mastectomy scar. Prior to the initiation of radiation therapy, the patient underwent a planning non-contrast CT scan in the supine position with the arms raised above her head. This initial image was used for strategic mapping and effective radiotherapy planning. 

**Figure 3:**
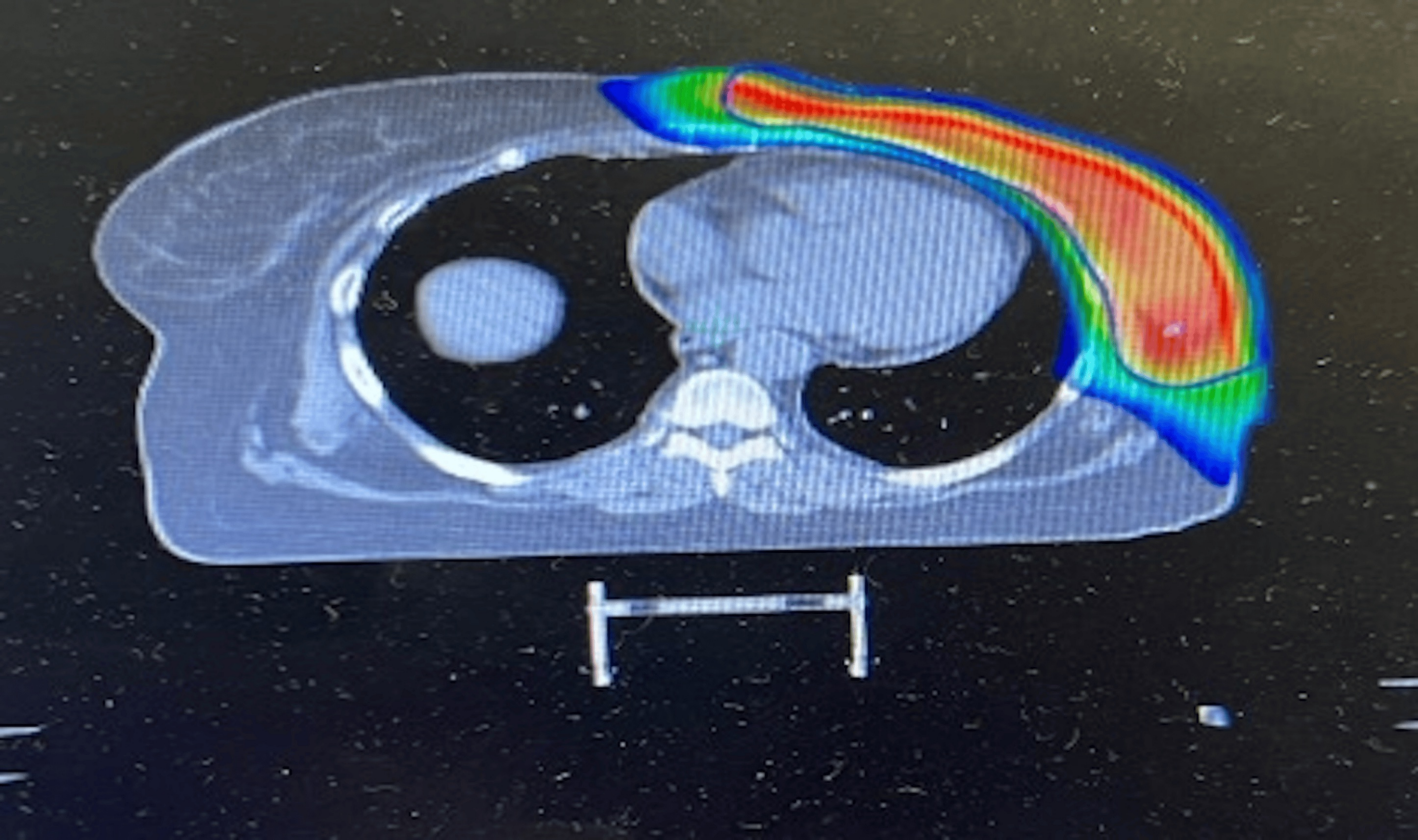
Radiation planning scan Axial dose distribution in supine position displaying the targeted region of post-mastectomy radiation therapy. The area of highest intended radiation dose is indicated by red; the area of lowest intended dose is indicated by blue.

Referring to Figure 3, the planning target volume is illustrated, encompassing the contour of the left breast along with an additional margin to accommodate setup variations and target motion. The heart, bilateral lungs, and contralateral breast have been identified as organs at risk, and the treatment plans were meticulously designed to minimize dose delivery to these sensitive structures. The patient received a total of 5800 centigray (cGy) at 200 cGy/fx delivered over 28 fractions to the entire left chest wall, in addition to 1000 cGy to the chest wall scar at 200 cGy/fx delivered over five fractions. 

## Discussion

MBC is a highly aggressive form of invasive breast cancer, often displaying resistance to therapeutic attempts. There is consistent clinical evidence associated with the pathologic nature of this disease. The majority of MBC cases demonstrate a triple-negative phenotype and an absence of expression of HER2 protein and present with a large primary tumor size and advanced disease at the time of diagnosis. Importantly, MBC has proven to display inferior survival rates when compared to non-metaplastic triple-negative breast cancers. One potential cause of the varied outcome in response is the unique heterogeneous morphology seen in MBC [1]. Presumably due to the unparalleled and rare character of MBC, there has been limited research performed regarding targeted treatment. Currently, MBC is managed according to the guidelines for other invasive breast cancers, consisting of a combination of chemotherapy, radiation therapy, and surgery. However, the molecular and histologic framework of MBC is distinctly varied from other cancer types; thus, the results of this approach have led to consistently poor prognosis. Under the current protocols, surgery demonstrates the most efficacious approach to disease management. According to a large study performed, patients may elect for either breast-conserving surgery (BCS) or mastectomy depending on the size of their primary tumor, as BCS has not been shown to result in inferior survival outcomes [3]. Individuals whose primary tumor size is greater than 5 cm are not considered to be appropriate candidates for BCS due to an increased risk of local recurrence. Although published research is limited on this disease due to its rarity, there are several factors that have been investigated and found to be associated with inferior disease prognosis. Interestingly, many elements of disease can vary significantly among racial and ethnic groups, suggesting a possible genetic component. A primary tumor size >50 mm, grade III+, and distant metastasis have also been associated with inferior survival rates. Among these prognostic factors listed, the one contributing most to discrepancies in survival among race and ethnic groups was found to be tumor stage at diagnosis. It is believed that socioeconomic factors such as insurance status and educational attainment level are influential characteristics in this inconsistency [2]. 

Beyond the variational outcomes among racial and ethnic groups, additional variations in outcomes have been observed between the various subtypes of MBC. As outlined by the World Health Organization, there are six subtypes of MBC: LGAC, SCC, SpCC, FLMC, MCMD, including chondroid, osseous, rhabdomyoid, and neuroglial components, and MMC. Among these, LGAC and FLMC have been associated with improved outcomes [4]. One hallmark molecular event that occurs in MBC is known as EMT. By way of this process, epithelial-like tumor cells lose their phenotypic cell adhesion molecules referred to as E-cadherins. This process gives way to the expression of mesenchymal cadherins through an event known as cadherin switching. Multiple transcription factors are known for orchestrating the changes in this molecular event and are considered to contribute to the established drug resistance of MBC. SNAIL, ZEB, and TWIST are transcription factors that are expressed to varying levels among the different subtypes of MBC. As a result of their varying presence, it can be presumed that there is a corresponding varying level of drug resistance among each subtype, which seemingly contributes to the inconsistencies noted in survival rates [4,5]. Additionally, a connection between EMT and the immune system has been revealed. The advantage of EMT in tumor survival lies in the unique capability of mesenchymal-like tumor cells to promote immunosuppression and facilitate immune evasion. Previous studies have demonstrated that tumors derived from mesenchymal phenotypic cell lines exhibit significantly reduced expression of MHC-I and elevated levels of programmed cell death ligand-1 (PD-L1) compared to tumors originating from epithelial phenotypic cell lines [6]. By secreting specific immunosuppressive factors, such as CD73, CSF1, and SPP1, mesenchymal cells are able to decrease both the number and the function of CD8+ T cells [6], thereby further enhancing the tumorigenic potential. Importantly, EMT does not produce cells that strictly display mesenchymal components, but rather this process creates novel cells that express a combination of both epithelial and mesenchymal markers. This complexity and variability in cell population are understood to contribute to the apoptotic resistance seen in tumor cells of MBC [8]. MBC presents a particularly complex and formidable therapeutic challenge, largely due to the limited efficacy of current therapies and the lack of optimized targeting strategies. Likely due to the rapid disease progression, studies have shown that MBC patients are more likely to receive mastectomy as primary surgical treatment, as opposed to triple-negative breast cancer patients who primarily receive lumpectomy as initial surgical intervention [9]. While surgical resection remains the primary intervention for MBC, there is increasing evidence to support the use of combination therapy with radiation and adjuvant chemotherapy. One study performed by Ullah et al. revealed that patients treated with a combination of surgery and adjuvant chemotherapy had the best overall outcome. Particularly, the use of combination surgery and radiation significantly improved the five-year survival rate (79%) compared to surgery alone (73.6%) [2]. As previously mentioned, MBC is characterized by a highly complex and distinctive molecular landscape. Key processes such as cadherin switching and PD-L1 overexpression have emerged as promising targets for the development of innovative therapeutic strategies. Preliminary evidence indicates that PD-L1 inhibitors, including pembrolizumab, may elicit favorable responses, highlighting a promising avenue for future targeted treatments [2]. 

## Conclusions

MBC is an exceedingly rare and highly aggressive subtype of invasive breast carcinoma characterized by its resistance to conventional therapeutic approaches. This case underscores the critical importance of comprehensive, multimodal treatment strategies and highlights the necessity for continued research into the molecular underpinnings and therapeutic vulnerabilities of MBC. Advancing our understanding through rigorous clinical and translational studies is essential for developing specific, evidence-based protocols that can improve prognosis and quality of life for patients afflicted with this formidable disease. 
